# The Probable, Possible, and Novel Functions of ERp29

**DOI:** 10.3389/fphys.2020.574339

**Published:** 2020-09-08

**Authors:** Margaret Brecker, Svetlana Khakhina, Tyler J. Schubert, Zachary Thompson, Ronald C. Rubenstein

**Affiliations:** ^1^Cystic Fibrosis Center, The Children’s Hospital of Philadelphia, Philadelphia, PA, United States; ^2^Department of Pediatrics, Perelman School of Medicine at the University of Pennsylvania, Philadelphia, PA, United States; ^3^Division of Allergy and Pulmonary Medicine, Department of Pediatrics, Washington University in St. Louis School of Medicine, St. Louis, MO, United States

**Keywords:** endoplasmic reticulum, ERp29, chaperone, escort, Golgi, secretory pathway, trafficking, COP II

## Abstract

The luminal endoplasmic reticulum (ER) protein of 29 kDa (ERp29) is a ubiquitously expressed cellular agent with multiple critical roles. ERp29 regulates the biosynthesis and trafficking of several transmembrane and secretory proteins, including the cystic fibrosis transmembrane conductance regulator (CFTR), the epithelial sodium channel (ENaC), thyroglobulin, connexin 43 hemichannels, and proinsulin. ERp29 is hypothesized to promote ER to *cis-*Golgi cargo protein transport *via* COP II machinery through its interactions with the KDEL receptor; this interaction may facilitate the loading of ERp29 clients into COP II vesicles. ERp29 also plays a role in ER stress (ERS) and the unfolded protein response (UPR) and is implicated in oncogenesis. Here, we review the vast array of ERp29’s clients, its role as an ER to Golgi escort protein, and further suggest ERp29 as a potential target for therapies related to diseases of protein misfolding and mistrafficking.

## Introduction

The endoplasmic reticulum (ER) is a membrane bound organelle present in eukaryotic cells. It is the site of co-translational synthesis of secretory proteins and membrane translocation of transmembrane proteins (Barlowe and Miller, 2013). The nascent proteins that are being synthesized and translocated into the ER lumen have unstable tertiary structures and heavily rely on interaction with ER luminal molecular chaperones, a group of proteins that assist in protein folding, to avoid misfolding and aggregation (Buck et al., 2007). Depending on the nascent luminal client, ER chaperones may stabilize a client through binding to the lumen-exposed hydrophobic regions of the nascent protein, catalyzing the formation of disulfide bridges, and/or by binding to the exposed glycosyl side chain that was added to the nascent amino acid chain upon its entry into the ER (lectin chaperones). With these functions as guidelines, ER luminal chaperones are often classified into four groups: folding chaperones, that assist with protein folding, including the Protein disulfide isomerase (PDI) and peptidyl-prolyl *cis/trans* isomerase (PPI) families; lectin chaperones, including calnexin and calreticulin; general heat shock or stress-responsive chaperones, including BiP/GRP78, GRP170, GRP94, and the non-classical molecular chaperones, including ERp29 and HSP47 (Mahdi et al., 2016). The lack or imbalance of chaperone function due to mutations that disrupt client interaction with chaperone(s), or transcriptional dysregulation, can lead to protein aggregation within the ER and initiation of the unfolded protein response (UPR) (Mahdi et al., 2016). Many ER chaperones have increased expression as a part of the UPR (Gomez-Navarro and Miller, 2016), but, if this initial response is not successful in resolving or eliminating protein aggregates, apoptosis pathways will be activated to eliminate the irreparably damaged cell (Mahdi et al., 2016).

Many secretory proteins require additional post-translational modifications in the form of glycosylation, phosphorylation, and protein cleavage in the Golgi Network (GN) to assume their final, active conformations (Potelle et al., 2015). After a secretory protein is fully synthesized and properly folded in the ER, it is exported to the *cis-*Golgi, commonly via the Coat Complex II (COP II) secretory vesicle pathway (Aridor et al., 1998), and ER escort chaperones are often packaged into the COP II vesicle together with their clients (Di Martino et al., 2019). In the *cis-*Golgi, secretory proteins may dissociate from the ER chaperone, potentially due to the more acidic pH of Golgi compartment compared to ER, and ultimately continue through the GN to undergo additional post-translational modifications and maturation (Gomez-Navarro and Miller, 2016). In contrast to the client protein, many of the ER chaperones are returned to the ER via the Coat Complex I (COP I) retrieval pathway (Gomez-Navarro and Miller, 2016). The majority of the luminal ER chaperones contain a characteristic C-terminal ER retention motif -K-D-E-L (Lys-Asp-Glu-Leu) (Pelham, 1990). This motif functions to enforce retrieval of these ER chaperones from the *cis-*Golgi via an interaction with the KDEL-Receptor (KDEL-R) and subsequent inclusion of this complex in COP I vesicles (Yamamoto et al., 2001). The current understanding of the KDEL-R role in the secretory protein pathway is limited to its role in vesicle transport by COP I. However, there are recent reports that observed KDEL-R at the plasma membrane of the cell (Becker et al., 2016), as well as data supporting the hypothesis that KDEL-R has a role in COP II-mediated ER→Golgi trafficking (Bikard et al., 2019).

The dysregulation of nascent protein folding within the ER, or dysregulation of protein transport from the ER to the GN, is characteristic of many protein folding diseases, including Cystic Fibrosis (CF), Alpha-1 Antitrypsin Deficiency, Alzheimer’s Disease, Parkinson’s Disease, Type 2 Diabetes (T2D), certain cancers, and others (Bartoszewski et al., 2008; Wang and Kaufman, 2012). CF is caused by mutations in the Cystic Fibrosis Transmembrane Conductance Regulator (CFTR) gene that encodes a chloride ion transporter typically found on the apical surface of epithelial cells (Welsh and Smith, 1993). CFTR biogenesis requires complex organization within the membrane and folding in the ER, as well as further processing in Golgi for CFTR to eventually assume its active form at the apical plasma membrane (Kim and Skach, 2012). The most common disease-causing CFTR mutation is F508del (an in-frame deletion of Phenylalanine 508), which primarily results in a mutant CFTR protein that is poorly folded and does not exit the ER (Lukacs and Verkman, 2012). Recent developments in therapy for CF, including those aimed at restoring proper biosynthesis of F508del, have dramatically improved clinical outcomes for people with CF (Lopes-Pacheco, 2019). One of the earliest small molecules that was demonstrated to improve F508del CFTR trafficking to the plasma membrane, and function is sodium 4-phenylbutyrate (Buphenyl^®^, 4PBA) (Rubenstein et al., 1997; Rubenstein and Zeitlin, 1998). In our group’s interrogation of the mechanism by which 4PBA acts, we identified that a non-classical ER chaperone ERp29 is upregulated in response to 4PBA treatment of CF bronchiolar epithelial cells (Suaud et al., 2011a). Further studies by our group demonstrated that ERp29 is required for proper biogenesis of wild type CFTR, and that overexpression of ERp29 can rescue the aberrant biosynthesis and trafficking of F508del CFTR (Suaud et al., 2011a). These data identify ERp29 as a novel drug target in a disease of aberrant protein folding and trafficking.

ERp29 is ubiquitously expressed throughout the different tissue types (Shnyder and Hubbard, 2002), especially in secretory tissues, and is implicated in the biogenesis of many proteins (Table 1). Our group’s work has demonstrated that ERp29 promotes CFTR maturation (Suaud et al., 2011a) and regulates the maturation of the epithelial sodium channel (ENaC) (Grumbach et al., 2014; Bikard et al., 2019) in epithelial cells. In addition, our group’s recent data have suggested a role for ERp29 in regulating proinsulin biosynthesis (Viviano et al., 2020). These data, and the potential general function of ERp29 suggested by its wide variety of putative clients (Table 1) further emphasizes the importance of understanding of the mechanism by which ERp29 contributes to the folding, trafficking, and function of these client proteins. In this paper we will provide a comprehensive analysis of ERp29 role as an essential ER to Golgi escort chaperone by highlighting the effect ERp29 has on client biogenesis, and by reviewing the potential role of ERp29 in client associated disease etiology.

**TABLE 1:** ERp29 clients and implications in disease.

ERp29 client	Function	Disease implications	References
Epithelial sodium channel (ENaC)	Epithelial sodium channel regulates airway surface liquid volume, sodium concentration, blood volume, blood pressure	Liddle’s syndrome, pseudohypoaldosteronism, hypertension	Grumbach et al., 2014
Cystic fibrosis transmembrane conductance regulator (CFTR)	ABC transporter of chloride ions in epithelia regulates sodium and water balance	Cystic fibrosis (CF)	Cormet-Boyaka et al., 2004; Suaud et al., 2011a
(Pro)insulin	Peptide hormone. Regulator of blood glucose levels	Type two diabetes (T2D)	Smeekens et al., 1992; Nishigori et al., 1996; Viviano et al., 2020
Thyroglobulin (Tg)	Thyroid hormone (T_3_ and T_4_) precursor	Goiter, hypothyroidism	Sargsyan et al., 2002a; Baryshev et al., 2006; Citterio et al., 2013
Alpha-1 antitrypsin (A1AT)	Serpin protein protects tissues from proteases	Liver failure, cirrhosis, chronic obstructive pulmonary disease (COPD)	Sifers et al., 1988; Davis et al., 1990; McElvaney et al., 2020
Alpha-1 type 1 collagen (Collagen-I)	Structural protein in bone, scelera, skin, and connective tissues	Osteogenesis imperfecta (OI), Ehlers-Danos syndrome (EDS)	Byers and Steiner, 1992; Myllyharju and Kivirikko, 2001; DiChiara et al., 2016; Miller and Grosel, 2020
Connexin-43 (Cx43)	Gap junction alpha-1 protein	Oculodentodigital dysplasia (ODDD)	Paznekas et al., 2003; Bao et al., 2004; Salameh, 2006; Laird, 2008; Das et al., 2009
PKR-like endoplasmic reticulum kinase (PERK, EIFA2K3)	Transcription factor phosphorylates α-subunit of EIF2 to repress transcription in ER stress conditions	Wollcott-Rallison syndrome	Julier and Nicolino, 2010; Farmaki et al., 2011
ATP-binding cassette transporter-1 (ABCA1)	ABC transporter of lipids. Lipid removal pathway component.	Tangier disease, high density lipoprotein (HDL) deficiency	Schmitz and Langmann, 2001; Sorrenson et al., 2013
ATP-binding cassette transporter-3 (ABCA3)	ABC transporter of lipids. Lipid removal pathway component.	Fatal respiratory distress syndrome (RDS), cataract microcornea syndrome, surfactant metabolism dysfunction type-3	Cheong et al., 2006; Beers and Mulugeta, 2017
Activating transcription factor-6 (ATF6)	Transcription factor. Transported to and cleaved in Golgi upon ER stress.	Cancer	Hirsch et al., 2014

### ERp29 Structure

ERp29 is a member of the ER luminal protein disulfide isomerase (PDI) chaperone family and contains four distinct domains (Ferrari et al., 1998). These include the N-terminal ER localization domain/signal sequence that is cleaved upon entry into the ER, the b-type PDI domain, the D-domain, and an ER retrieval KEEL (Lys-Glu-Glu-Leu) domain at the C-terminus. Although ERp29 contains a conserved thioredoxin-like PDI domain, it is classified as a “non-classical” chaperone as this domain, and holo-ERp29, contains only a single Cysteine and does not possess any thioredoxin enzymatic activity (Ferrari et al., 1998; Galligan and Petersen, 2012). Nevertheless, ERp29 has several hydrophobic protein binding domains on its surface, and facilitates protein folding and ER export by interacting with multiple clients and other chaperones in the ER lumen (Barak et al., 2009; Nakao et al., 2017).

Insight into the structural and functional domains of human ERp29 can be inferred from studies of homologs and paralogs from other species, including rat and mouse ERp29, and *Drosophila melanogaster* Windbeutel (Wind). Wind expression in *Drosophila* is essential for the transport of Pipe, a glycosaminoglycan-modifying enzyme with homology to heparan sulfate 2-*O*-sulfotransferase in mammals (Sergeev et al., 2001), to the Golgi where it regulates dorsal-ventral embryogenesis of *D. melanogaster* embryos via an extracellular serine protease cascade (Sen et al., 2000). When Pipe is expressed in tissues without Wind, Pipe remains in the ER and embryo dorsal-ventral patterning does not occur; this results in embryonic death (Sen et al., 2000). Unlike mammalian ERp29, the *D. melanogaster* Wind protein has a conserved (C-X-X-C) PDI domain (Ma et al., 2003). The important structural and functional homology of the C-terminal D-domain between Wind and human ERp29 (Ma et al., 2003), allowed for better characterization of ERp29 function in earlier studies.

**FIGURE 1:**
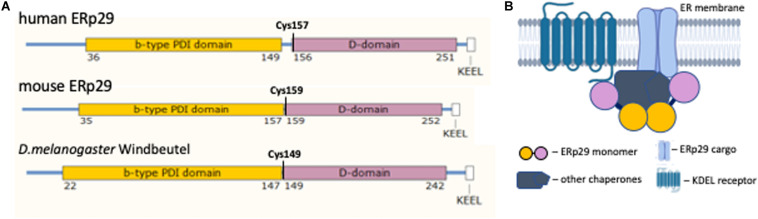
ERp29 structure. **(A)** The structural alignment of ERp29 protein functional domains from human, mouse, and *Drosophila melanogaster*. **(B)** Cartoon of the proposed ERp29 complex at the ER membrane (partially created with BioRender.com). The color scheme between **(A,B)** is consistent, demonstrating ERp29 dimerization at the N-terminus domain interface and serving as a linker of a larger protein complex.

The generation of high resolution crystal protein structure of human ERp29 was delayed by increased domain mobility in the structure due to the presence of a longer linker between N-terminal b-domain and the C-terminal D-domain in human compared to mouse and *D. melanogaster*, 7 versus 2 amino acids, respectively (Figure 1A) (Barak et al., 2009). All mammalian ERp29 proteins lack a classical thioredoxin (C-X-X-C) motif and therefore lack such thioredoxin function (Ferrari et al., 1998; Ma et al., 2003). At the same time the *in vitro* and *in vivo* studies of ERp29 protein–client interactions demonstrated that the D-domain located in the C-terminal half of ERp29 is highly conserved between species and required for client binding and processing (Lippert et al., 2007). *In vitro* studies of Wind demonstrated that this chaperone has binding affinity for clients with Tyrosine (Y) and Phenylalanine (F) aromatic residues, either adjacent (e.g., –(F,Y)-(F,Y)-) or separated by a single amino acid (e.g., –(F,Y)-X-(F,Y)-) (Barnewitz et al., 2004). A similar client binding affinity has been suggested for mammalian ERp29 (Barak et al., 2009).

The hydrodynamic properties of human ERp29 suggest that the protein assumes a homodimeric tertiary structure (Hermann et al., 2005). This homodimerization occur through an interaction of two N-terminal ERp29 domains (Barak et al., 2009). In *D. melanogaster*, Wind dimerization creates a negatively charged acidic cleft; this cleft is required for client interaction (Barnewitz et al., 2004). However, the cleft formed by homodimerization of the b-domain of mammalian ERp29 lacks similar biochemical characteristics, and cannot bind Wind clients (Hermann et al., 2005). That said, ERp29 dimerization is essential for its function as an ER chaperone (Rainey-Barger et al., 2007). Further characterization of the ERp29 dimer cleft is required to further elucidate its function as a chaperone and escort protein.

The tightly bound, non-globular, dimeric structure of ERp29 places the two C-terminal D-domains on the opposite sides of an elongated complex (Figure 1B) (Hermann et al., 2005). This structure suggests that the ERp29 homodimer may serve as a linker that coordinates a larger hetero-complex of chaperones, its clients, and ER export machinery (Meunier et al., 2002). The proper folding of the D-domain is essential for ERp29 function (Lippert et al., 2007). The N-terminal and C-terminal domains of ERp29 are joined with a hydrophobic linker. This linker shares a conserved Cysteine residue at the beginning of the D-domain. Mutation of this residue (human C157S) affects hydrophobic characteristics of the linker and disrupts the function of ERp29 homodimer (Hermann et al., 2005). In our discussion below, we will address the functional effects of this C157S mutation on client protein binding and chaperone function *in vivo*.

### Shuttling of ERp29 Between the ER and Golgi

**FIGURE 2:**
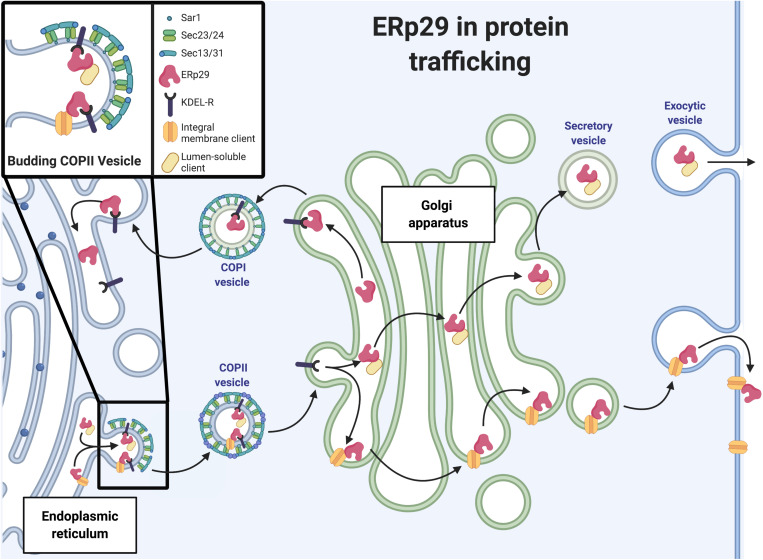
ERp29 in protein trafficking. Schematic representation of ERp29 role in ER to Golgi secretory pathway (image created with BioRender.com).

The retrograde and anterograde vesicular transport of cargo proteins between the ER and the *cis*-Golgi network is controlled by COP I and COP II machinery, respectively (Figure 2). COP II trafficking is of particular relevance to secretory protein biogenesis as it is major trafficking machinery of the early secretory pathway. The COP II complex, or COP II machinery, is comprised of the heteromeric protein complexes Sec23-Sec24 and Sec13-Sec31, the assembly and disassembly of which is regulated by the Sar1 GTPase (Figure 2). Sar1-GDP is activated to Sar1-GTP via Sec12, a transmembrane guanine nucleotide exchange factor (GEF) located on the luminal face of the ER membrane. It is constitutively active, as it displays GEF activity in reactions containing only Sar1, GTP, and synthetic phospholipids, though its GEF activity can be enhanced by potassium (Futai et al., 2004; McMahon et al., 2012). For larger secreted cargo (e.g., collagen), Sec12 is concentrated at the site of COP II vesicle formation. In the specific case of collagen, Sec12 is concentrated at the site by cutaneous T-cell lymphoma-associated antigen 5, or cTAGE5 (a component of the collagen cargo receptor) to increase nucleotide exchange on Sar1 (Saito et al., 2014). From there, Sar1-GTP and Sec23-Sec24 interact to recruit the additional cargo proteins to the emerging COP II vesicle. Sec24 has four isoforms and is the main cargo recognition adaptor of COP II machinery; these cargoes often contain recognition motifs including di-acidic motifs (e.g., D-X-E, D-X-D) or large C-terminal di-hydrophobic or di-aromatic motifs (e.g., FF, YY, and/or FY); these motifs are crucial for cargo exit from the ER via COP II vesicles (Votsmeier and Gallwitz, 2001; Barlowe, 2003). Sec13-Sec31 then binds to the growing COP II unit and causes the budding of the COP II vesicle. The Sec13-Sec31 complex also recruits other Sar1/Sec23-Sec24 complexes to the budding vesicle, which further concentrates proteins targeted for ER exit. At the *cis*-Golgi, Sec23 promotes hydrolysis of GTP by Sar1 and return to its inactive Sar1-GDP form; this facilitates disassembly of the COP II complex.

The KDEL receptor (KDEL-R) regulates the retrograde trafficking of proteins containing a C-terminal ER retention signal from the *cis-*Golgi to the ER via COP I transport machinery. KDEL-R has three isoforms (1, 2, and 3) that are G-protein coupled-like receptors of the PQ-loop superfamily with seven transmembrane spanning helices (Giannotta et al., 2012; McMahon et al., 2012). These receptors primarily interact with C-terminal -K-D-E-L motifs in higher eukaryotes, or -H-D-E-L motifs in yeast. This process is pH dependent, with the low pH of the Golgi (∼6) enhancing the interaction between the KDEL-R and the KDEL motif; the relatively neutral pH of the ER (∼7.2–7.4) promotes release of the protein from the KDEL-R (Scheel and Pelham, 1996; Gomez-Navarro and Miller, 2016; Bräuer et al., 2019). Presumably, to fulfill this function, the KDEL-R must get to the *cis-*Golgi via COP II transport. However, a function for the KDEL-R in this anterograde movement is less well-demonstrated. Furthermore, there are no well-defined unique functions for the different isoforms of KDEL-R, although some recent data suggesting KDEL-R2 and KDEL-R3 play a role in controlling the UPR (Trychta et al., 2018). KDEL-R2 and KDEL-R3, but not KDEL-R1, were found to have increased expression in SH-SY5Y, INS-1, 832/13, HEK293, and rat primary cortical neurons after Ca2 + depletion of the ER using thapsigargin and after ER stress induction by tunicamycin. Additionally, KDEL-R2 and KDEL-R3 expression was increased after addition of the transcription factor XBP1, a known regulator of ER stress proteins (e.g., Erdj4) in HEK293 cells; *in silico* studies also suggested putative binding sites for XBP1 on the promoters for KDEL-R2 and -R3, but not -R1. There are also some data suggesting a role for KDEL-R1 in protein recognition and endocytosis on the plasma membrane in HeLa cells (Becker et al., 2016; Trychta et al., 2018). KDEL-R1 was found at the cell surface and was shown to be crucial in allowing fluorescent markers with an -H-D-E-L tag to gain entry into the cytoplasm. Taken together, these data further suggest a role for KDEL-R at sites in the secretory pathway more distal to the *cis*-Golgi.

With regards to the KDEL-R having a role in anterograde ER→Golgi trafficking via COP II, our group has recently found that ERp29 likely interacts with KDEL-R1 as part of the anterograde trafficking of the epithelial sodium channel (ENaC) in CFBE41o- CF bronchial epithelial cells and MDCK renal epithelial cells (Bikard et al., 2019). Furthermore, we demonstrated that deletion of ERp29’s C-terminal -K-E-E-L ER retention motif (a KDEL homolog) reduced the interaction of ENaC with the Sec24D as seen in co-immunoprecipitation experiments (CFBE41o- and MDCK cells). Similar effects on ENaC biogenesis were observed with depletion of KDEL-R1 or Sec24D (Bikard et al., 2019). Taken together, these data suggest ERp29, through an interaction with KDEL-R1, regulates ENaC biogenesis, which indicates a novel role for KDEL-R1 in anterograde protein trafficking.

As mentioned above, ERp29 contains a C-terminal KEEL motif that interacts with the KDEL-R rather than the more classical C-terminal KDEL motif. KEEL-harboring ER constituents are less fully retained in the ER than those with KDEL; in other words, KEEL binding has lower affinity than KDEL to KDEL-R in the acidic Golgi (Giannotta et al., 2012; Stevens et al., 2019). In fact, our group has found ERp29 in more distal compartments, including at the surface of epithelial cells and secreted into culture media in CFBE41o- cells (Suaud et al., 2011a), suggesting that ERp29 may have other functional roles beyond promoting anterograde ER → Golgi trafficking of client cargo via KDEL-R and COP II machinery.

In addition to KEEL, there are other KDEL-like motifs described in the literature. Some evidence suggests that the KDEL-R isoforms preferentially bind to different KDEL-like motifs, with KDEL-R2 having a more narrow specificity than KDEL-R1 or -R3 (Raykhel et al., 2007). In addition, Raykhel et al. (2007) demonstrated that 45 different KDEL-like motifs resulted in ER retention in HeLa cells; when any of these KDEL-like motifs were not present, predominant localization to the Golgi was the almost exclusive result. Interestingly, a KXEL motif caused almost exclusive localization in the ER, with the “X” amino acid being polar, charged, or hydrophobic.

### ERp29 and the ER Stress Response

As with other ER resident chaperones such as BiP/Grp78, ERp29 expression is upregulated during ER stress (ERS) and the unfolded protein response (UPR) in both pancreatic beta cells and thyroid epithelial cells (Sargsyan et al., 2004; Gao et al., 2016); however, its role in ERS and UPR remains somewhat unclear (Farmaki et al., 2011). That ERp29 expression is elevated in some tumors or when genotoxic stress is induced with ionizing radiation (Zhang et al., 2008; Farmaki et al., 2011) may suggest a broader role for ERp29 in stress conditions, and perhaps in apoptosis. Additionally, ERp29 is upregulated by treatment with doxorubicin, an anti-neoplastic chemotherapeutic drug, in a p53-dependent manner in PC3 prostate cancer cells and Mouse Embryonic Fibroblasts (Farmaki et al., 2011). However, upregulation of ERp29 expression is also observed when ERS is present in both thyroid cells and INS1 pancreatic β-cells (Sargsyan et al., 2004; Gao et al., 2016), and in lactating mammary gland cells compared to resting glands (Mkrtchian et al., 2008). It is thus hypothesized that ERp29’s involvement in UPR is limited to its role as a chaperone for secretory proteins (Sargsyan et al., 2004; Mkrtchian et al., 2008; Farmaki et al., 2011), which is consistent with ERp29 being most highly expressed in secretory cells (Sargsyan et al., 2002a). This role for ERp29 in ERS is consistent with the hypothesis that ERp29 is critical in facilitating the exit of clients from the ER via COP II vesicles.

**FIGURE 3:**
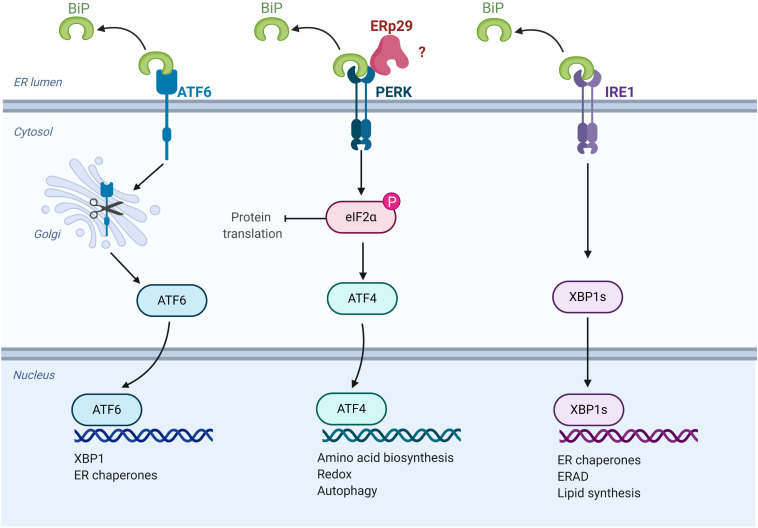
ERp29 role in the unfolded protein response (UPR). Schematic representation of UPR pathways, highlighting the potential site of ERp29 action (image created with BioRender.com).

Under conditions of ERS, the UPR is initiated by the activation of three proteins that are both sensors of ER stress and regulators of the subsequent response: the transcription factor, ATF6, and two kinases, IRE1 and PERK (Figure 3) (Malhotra and Kaufman, 2007). Under resting circumstances, ATF6, IRE1, and PERK associate with and are “sequestered” in the ER by BiP/GRP78. In the presence of unfolded protein in the ER, BiP/GRP78 preferentially binds to the unfolded protein, thereby releasing ATF6, IRE1 and PERK to activate the UPR. The subsequent actions of these three sensor proteins when released from BiP/GRP78 lead to downregulation of protein translation and increased expression of key chaperones and pro-survival factors implicated in ERS, including BiP/GRP78 itself (Malhotra and Kaufman, 2007). Specifically, PERK phosphorylates and activates the translation-inhibiting protein, eIF2α, resulting in a global decrease in protein translation (Malhotra and Kaufman, 2007).

While it is logical to hypothesize that ERp29 is predominantly upregulated during ERS to facilitate stabilization of its clients for their eventual exit from the ER, there are also data suggesting that ERp29 may play a role in the underlying pathways that regulate and facilitate UPR and ERS. For example, siRNA-mediated knockdown of ERp29 during induced ERS was associated with decreased expression of BiP mRNA in INS1 rat pancreatic beta cells (Gao et al., 2016). Furthermore, ERp29 associates with PERK in co-precipitation studies (Farmaki et al., 2011; Gao et al., 2016), and increased ERp29 expression results in upregulated intracellular PERK levels, as well as increased phosphorylation/activation of its downstream target, eIF2α. On the other hand, overexpression of the ERp29 C157S mutant did not result in a similar increase of PERK levels, and interestingly, decreased the activation of eIF2α as compared to non-transfected controls (Farmaki et al., 2011). Taken together, these data suggest a direct role for ERp29 in UPR regulation that may be dependent on its interaction with PERK, perhaps by facilitating the interaction of PERK with its downstream client, eIF2α (Farmaki et al., 2011; Gao et al., 2016). Future studies should aim to further elucidate ERp29’s role in UPR, as this response plays a key role in the underlying pathophysiology of cancer and many other diseases, including those of secretory cells such as the insulin-secreting β-cells in Type II diabetes (Ozcan et al., 2004; Sundar Rajan et al., 2007). Additionally, it is currently unclear how *erp29* expression is regulated during ERS and UPR. Future work should aim to identify potential ER stress responsive elements in the *erp29* promoter or IRES sequences that allow preferential translation of *erp29* mRNA during ERS.

### ERp29 and Transmembrane Protein Biogenesis

As mentioned above, our interest in ERp29 derived from studies of the mechanism by which 4PBA rescues F508del CFTR trafficking in CF epithelia (Rubenstein et al., 1997; Rubenstein and Zeitlin, 1998, 2000; Rubenstein and Lyons, 2001; Suaud et al., 2011a, b). Upon identification of ERp29 potentially playing a role in this process, we extended these studies to test the role of ERp29 in the biogenesis of ENaC (Grumbach et al., 2014; Bikard et al., 2019), another epithelial ion channel relevant to CF. In this section, we will discuss ERp29’s role in regulating the transmembrane proteins biogenesis.

### Epithelial Sodium Channel (ENaC)

ENaC is a heterotrimer comprised of three similar subunits: α, β, and γ; alternate δ-ENaC channels found in some epithelia substitute a δ subunit for the α subunit (Giraldez et al., 2012). The three subunits presumably assemble in the ER before they are transferred to the Golgi by means of COP II machinery for maturation of N-glycosylation and furin-mediated cleavage of the α and γ subunits; the cleavage process may occur in the Golgi or later compartments. From there, the protein complex is brought to the plasma membrane where the channels play an important role in Na^+^ resorption in various epithelia, including the airway and renal collecting duct. Full cleavage of the α and γ subunits is associated with the channel having a high (∼1.0) open probability. Interestingly, some ENaC bypasses these Golgi modifications and arrives at the plasma membrane via an alternative pathway in a core glycosylated, uncleaved, low (∼0) open probability form. The most common clinical condition associated with ENaC is Liddle’s syndrome, a disease caused by constitutively active ENaC that has impaired retrieval from the apical plasma membrane (Schild et al., 1995). ENaC hyperactivity is also seen in cystic fibrosis patients as a result of its dysregulation caused by mutations in the cystic fibrosis transmembrane regulator (CFTR) protein (Kunzelmann et al., 2001).

We demonstrated that the Sec24D cargo recognition component of COP II machinery plays a pivotal role in directing ENaC from the ER to the Golgi for further processing in CFBE41o- and MDCK cells (Grumbach et al., 2014). Sec24D likely interacts with the C-terminus of α-ENaC, as deletion of a 5 amino acid sequence (R-S-R-Y-W) within this region prevents ENaC from leaving the ER; removal of the C-termini of the β and γ subunits still allows exit of ENaC from the ER (Burrows et al., 2000; Mueller et al., 2007). Similarly, we demonstrated that depletion of Sec24D protein expression reduces the number of cleaved subunits on the cell surface but does not reduce the amount of β-ENaC at the cell surface. These data suggest that when the COP II ER exit pathway is blocked, ENaC can still reach the plasma membrane by a non-COP II pathway that allows the channel to bypass cleavage by furin in the Golgi or later compartments (Bikard et al., 2019).

Interestingly, ERp29 also appears to direct ENaC to the Golgi for cleavage of its α and γ subunits during biogenesis, as both depletion of ERp29 protein expression and expression of a mutant ERp29 with its single cysteine changed to serine (ERp29 C157S) have the same effect as depletion of Sec24D (Grumbach et al., 2014). Similarly, our group’s more recent data suggest that an interaction of ERp29 with the KDEL-R is required to promote transport of ENaC through COP II vesicles from the ER to the Golgi to facilitate the cleavage of α- and γ-ENaC (Bikard et al., 2019). Both deleting ERp29’s C-terminal KEEL motif, which should interrupt ERp29’s interaction with KDEL-R, and depletion of the expression of KDEL-R1 decreased both association of β-ENaC with Sec24D and levels of cleaved, active ENaC at the cell surface, again without altering total ENaC at the cell surface. Together, these data from our group suggest that ERp29, at least in part through an interaction with KDEL-R1, promotes inclusion of nascent ENaC into COP II vesicles for transport to the Golgi where α and γ undergo cleavage by furin in the *trans-*Golgi or later compartments. It is also possible that, because its KEEL ER retention motif that has lower affinity for the KDEL-R than KDEL in the *cis*-Golgi, ERp29 more readily escapes ER retention by dissociating from KDEL-R in the *cis*-Golgi, and then accompanies or escorts ENaC to the *trans-*Golgi to “directly” facilitate ENaC cleavage (Gomez-Navarro and Miller, 2016; Bräuer et al., 2019). This would provide one hypothetical mechanism by which ENaC that is not associated with ERp29 could bypass such Golgi processing.

### Cystic Fibrosis Transmembrane Conductance Regulator (CFTR)

As is typical for N-glycosylated transmembrane proteins, including ion channels, CFTR is transported from the ER to the Golgi with “immature” high mannose-type N-linked glycans; “maturation” of these glycans then occurs in the Golgi by enzymatic modification. For CFTR, this maturation is biochemically assessed as a change from “band B,” having Endoglycosidase H-sensitive N-linked glycosylation indicative of processing in the ER, to a higher molecular weight “band C” harboring glycans that have been modified in the Golgi and are now resistant to release with Endoglycosidase H (Cormet-Boyaka et al., 2004). The F508del CFTR mutant protein does not undergo this normal glycosyl maturation, indicative of impaired ER→Golgi transport, and does not reach the apical surface of epithelia (Cheng et al., 1990; Riordan, 1999). F508del also has reduced interaction with the Sec24D cargo recognition component of COP II ER exit machinery in HEK293 cells (Wang et al., 2004), further suggesting that its ER→Golgi transport is impaired. F508del that does not exit the ER is degraded via ubiquitination and ER associated degradation (ERAD) (Ward et al., 1995; Lukacs and Verkman, 2012).

Our group’s earlier studies of the mechanism by which 4-phenylbutyrate (4PBA) rescues F508del trafficking and function in IB3-1 CF bronchiolar epithelial cells concentrated on 4PBA’s regulation of the cytosolic 70 kDa heat shock protein chaperones Hsc70 and Hsp70 (Yang et al., 1993; Rubenstein and Zeitlin, 2000; Rubenstein and Lyons, 2001; Suaud et al., 2011b). However, a primarily cytosolic mechanism of action for the effects of 4PBA would seemingly not be applicable to primarily ER luminal cargoes such as PiZ Alpha-1 antitrypsin (A1AT) where 4PBA also has positive effects in model systems (see discussion below) (Burrows et al., 2000; Greene and McElvaney, 2010). Thus, when we found that 4PBA also caused increased ERp29 mRNA and protein expression (Suaud et al., 2011a), we began to focus on this non-classical ER chaperone as potentially mediating a common mechanism for improvement in trafficking of proteins that are retained in the ER.

In fact, our group’s work demonstrates that wild type CFTR maturation from the ER through the Golgi and its function at the cell surface can be reduced by depleting ERp29 expression in IB3-1 cells and T84 colonic adenocarcinoma cells; these data were the first to describe a positive role for an ER chaperone in CFTR biogenesis. Furthermore, we demonstrated that overexpression of ERp29 can rescue F508del trafficking and restore its expression at the surface of epithelial cells (Suaud et al., 2011a). Interestingly, we found that ERp29 also traverses this vesicular trafficking/secretory pathway, as it is found both at the cell surface and in the conditioned media, suggesting that ERp29 may accompany or escort clients through more distal compartments (Suaud et al., 2011a).

CFTR contains exactly one putative ERp29 interaction motif on its luminal extracellular face, ^1013^P-Y-I-F^1016^, on extracellular loop 5. Furthermore, there are naturally occurring disease-causing mutations within or adjacent to this motif described (Y1014C, F1016S, P1013H, and P1013L) (Cystic Fibrosis Centre at the Hospital of Sick Children in Toronto, 2020). These mutations hypothetically lack the necessary or appropriately conformed (in the case of the P1013 mutation at the end of transmembrane helix 9) site for interaction with ERp29 and, thus, may be unable to exit the ER.

### Other Transmembrane Proteins and ERp29

ERp29 may be important in trafficking of other plasma membrane channel or transporter proteins besides ENaC and CFTR. This hypothesis is supported by ERp29 having abundant expression in secretory cells (Shnyder and Hubbard, 2002) (specifically related to channel proteins), as well as having increased expression when cells are treated with 4PBA, a treatment that can also rescue the impaired biogenesis of other mutant transmembrane transport proteins.

For example, 4PBA increases surfactant protein secretion caused by mutations in the lipid transporter proteins ATP binding cassette A1 (ABCA1) and A3 (ABCA3) in HEK293 cells (Cheong et al., 2006; Sorrenson et al., 2013). Mutations of the ABCA1 and ABCA3 lipid transporters that cause their retention in the ER can be rescued with 4PBA at similar concentrations to that used to rescue F508del CFTR. As CFTR is also a member of the ABC transporter family (CFTR is ABCC7), it is logical to hypothesize that the rescue of ABCA1 and ABCA3 by 4PBA may result from a similar mechanism involving increased ERp29 expression. Similarly, a mutation in the bile salt export pump BSEP/ABCB11 that causes ER retention is also rescued by 4PBA in MDCK cells (Hayashi and Sugiyama, 2007). Similar effects of 4PBA are seen in neuronal cells, with 4PBA rescuing misfolded creatine transporter-1 (CTR-1) channels from the ER and allowing their trafficking to the cell surface in HEK293 cells (El-Kasaby et al., 2019). Finally, 4PBA also restores cell surface expression of monocarboxylate transporter 8 (MCT8, a thyroid hormone transporter) in patients with Allan-Herndon-Dudley syndrome, a condition characterized by mutations in MCT8 resulting in retention of the transporter in the ER (Braun and Schweizer, 2017).

Taken together, the similarities of F508del CFTR and the trafficking mutations of these other mutant transporters that are rescued by treatment with 4PBA, and 4PBA’s known effect of increasing ERp29 mRNA and protein levels, suggests the hypothesis that ERp29 may play a common role in the biogenesis of these transmembrane channel/transporter proteins. Significant work will be required to more thoroughly test this hypothesis.

### ERp29 Role in Secretory Protein Biosynthesis

As a chaperone of the ER lumen, ERp29 is demonstrated to associate with and influence the biosynthesis of a number of diverse secretory proteins. However, the mechanism(s) by which ERp29 exerts these effects are not yet well-delineated. In this section we review ERp29’s described clients that are secreted, as well as glean mechanistic insight from these data.

### Thyroglobulin (Tg)

Thyroglobulin (Tg) is a highly abundant iodinated glycoprotein in the follicular thyrocytes of the thyroid gland, and is the precursor to the thyroid hormones thyroxine (T_4_) and triiodothyronine (T_3_) (Pirahanchi et al., 2020). The biosynthesis of Tg is complex, and several ER chaperones form heterocomplexes with Tg in the thyrocyte ER. The ER chaperones involved in Tg biosynthesis include ERp72, GRP94, BiP/GRP78, PDI, calnexin, and ERp29 (Kuznetsov et al., 1994; Sargsyan et al., 2002b; Park et al., 2005). Focusing specifically on ERp29, immunoprecipitation of Tg from FRTL-5 thyrocytes treated with chemical crosslinking reagents revealed Tg association with ERp29, as well as with BiP/GRP78 and GRP94 (Sargsyan et al., 2002b). Interestingly, ERp29 overexpression itself increased expression of other Tg folding complex members, including BiP/GRP78, calnexin, and PDI, suggesting a key role for ERp29 in the Tg protein folding complex that promotes Tg processing.

TSH (thyroid stimulating hormone that promotes T_3_ and T_4_ secretion) increases the expression of ERp29 (Kim and Arvan, 1993). Consistent with this, ERp29 and Tg colocalize in both FRTL-5 and PCCL3 rat thyroid cells, and overexpression of ERp29 in these cells increases Tg secretion nearly two-fold while siRNA mediated knockdown of ERp29 reduces Tg expression and arrests Tg secretion (Kwon et al., 2000; Sargsyan et al., 2002b). In contrast, BiP/GRP78 or GRP94 overexpression decelerates the maturation and folding of Tg in the ER and consequently decreases Tg secretion (Muresan and Arvan, 1997).

Additional evidence for ERp29’s central role in Tg biosynthesis comes from studies where mutation of ERp29’s conserved single Cysteine residue, C157, to Alanine (C157A) or serine (C157S) greatly reduced Tg secretion levels (Baryshev et al., 2006). Similarly, mutation of a tyrosine residue conserved between ERp29 and Wind (Y64S in ERp29) also reduced Tg secretion, as did mutation of a solvent exposed Glutamine residue, Q70 (Q70A) (Ma et al., 2003; Baryshev et al., 2006).

Over 100 naturally-occurring mutations of the *TG* gene are known with an incidence of approximately 1 in 100,000 newborns (Targovnik et al., 2011; Citterio et al., 2013; Siffo et al., 2018). Manifestations of these mutations include congenital goiter, low serum Tg, high serum TSH, and hypothyroidism (Targovnik et al., 2010). The most severe Tg-mutant disorders are associated with Tg misfolding and sequestration within the ER (Citterio et al., 2013; Morishita et al., 2020), including some Tg mutations that cause defects in the export of Tg from the ER (Kim et al., 1996; Rubio and Medeiros-Neto, 2009; Targovnik et al., 2010). While the precise location of ERp29 binding on Tg is not known, Tg has several -(F,Y)-X-(F,Y) and -(F,Y)-(F,Y)- motifs that are consistent with other ERp29 clients (Barak et al., 2009), such as CFTR and ENaC. However, there are currently no reported naturally occurring mutations in Tg that disrupt the proposed hydrophobic binding sites that ERp29 prefers in its other clients.

Given that upregulation of ERp29 increases the secretion of Tg, enhancement of ERp29 function may be a potential target for treatment of mutant-Tg associated disorders. Just as our group has previously used sodium 4-phenylbutyrate (4PBA) as a small molecule for F508del CFTR rescue (Suaud et al., 2011b), 4PBA may be similarly able to rescue Tg secretion by increasing ERp29 abundance. Future studies are needed to identify other ERp29-centric therapies that rescue misfolded Tg and promote its secretion.

### Alpha-1 Antitrypsin (A1AT)

Alpha-1 antitrypsin (A1AT) is a secreted protease inhibitor that impedes the unfettered action of serine proteases, most notably neutrophil elastase in the lung. A1AT is encoded by *SERPINA1*, and mutations in this gene result in A1AT deficiency. The most common A1AT mutant, PiZ (E342K), causes A1AT retention in the ER due to misfolding and polymerization of the nascent protein (Sifers et al., 1988; Davis et al., 1990). The polymerization of PiZ causes expansion of the ER, primarily in hepatocytes, and reduced mobility of ER luminal chaperones, which may impair their functionality; in some circumstances, this results in hepatocyte death (Ordóñez et al., 2013). Another A1AT mutant, Null Hong Kong (NHK, S343Rfs), also does not exit the ER but does not polymerize and is instead subject to degradation by ER associated degradation (ERAD) machinery (Zhong et al., 2015). In contrast to PiZ, NHK has no effect on the mobility of ER luminal chaperones (Davis et al., 1990).

Disease due to A1AT deficiency primarily manifests in liver and lung. In the liver, aggregation and polymerization of the PiZ mutant in the hepatocyte ER may culminate in hepatocyte death, liver failure and cirrhosis as early as the newborn period (Patel and Teckman, 2018). Lack of protection from neutrophil elastase in the lung with A1AT deficiency increases lung susceptibility to injury, often manifesting clinically as emphysema or chronic obstructive pulmonary disease (COPD) (McElvaney et al., 2020).

Because PiZ A1AT is sequestered in the ER due to misfolding, as is F508del CFTR (Cheng et al., 1990; Rubenstein et al., 1997), the use of 4PBA to rescue PiZ secretion was explored. In fact, 4PBA improves the secretion of PiZ A1AT in both animal models and cellular systems (Burrows et al., 2000). However, this finding did not translate to significant clinical efficacy in one small pilot human study using 4PBA as a rescue therapy (Teckman, 2004). Even so, the preclinical data may suggest a mechanistic commonality of 4PBA action on F508del and PiZ through an increase in ERp29. Interestingly, A1AT contains a ^188^Y-I-F-F^191^ motif and preliminary data from our laboratory suggest ERp29 and A1AT may interact via this binding domain in co-precipitation experiments in Madin-Darby Canine Kidney (MDCK) and Cystic Fibrosis Bronchial Epithelial (CFBE41o-) cells (unpublished data). ERp29’s role as a regulator of A1AT biogenesis in the ER should be explored in the future to test the hypothesis that ERp29 exercises a significant effect on A1AT, and PiZ, protein folding, as well as subsequent liver and lung pathology.

### Collagen-I

ERp29 was recently shown to have a novel protective function in Type-I Collagen (collagen-I) synthesis. Collagen-I is the most abundant collagen protein in humans and is the primary structural component for skin and bone tissue (Shoulders and Raines, 2009). Mutations in the collagen-I gene, *COL1A1*, result in various collagenopathies, most notably osteogenesis imperfecta (OI) and Ehlers-Danlos syndrome (EDS). OI and EDS manifest as weakened bone structure and weakened or lax connective tissue, respectively (Byers and Steiner, 1992; Miller and Grosel, 2020). Notably, ERp29 has not been implicated as a factor in diseases of collagen-I or related processes to date.

Collagen-I is heavily modified in both the ER and the Golgi prior to secretion into the extracellular matrix. Recent data implicate ERp29, ERp44, PDIA3, PDIA4, and EroL1 as components of collagen-I’s proteostatic network in the ER (DiChiara et al., 2016). While complex, collagen-I biosynthesis includes the critical steps of the hydroxylation of various proline and lysine residues (Yamauchi and Sricholpech, 2012; Miller and Grosel, 2020). These hydroxylysines serve as attachment sites of oligosaccharides and as sites of covalent crosslinking to provide collagen-I it’s fibril strength (Yeowell and Walker, 2000), and mutations in several ER-hydroxylases cause specific OI and EDS disorders (Ha-Vinh et al., 2004; Yamauchi and Sricholpech, 2012). These ER-resident hydroxylases require ascorbate for proper function (Tajima and Pinnell, 1982; Franceschi et al., 1994) and non-hydroxylated collagen-I is retained in the ER (Miller and Grosel, 2020). Therefore, the presence of ascorbate promotes proper collagen-I processing in the ER while in ascorbate deficient conditions, the immature protein remains in the ER.

ERp29 may function to stabilize immature collagen-I in the ER until it can be hydroxylated. Depletion of ERp29 does not affect collagen-I secretion in ER-export conditions in the presence of sufficient ascorbate. In contrast, depletion of ERp29 decreases intracellular collagen-I and increases collagen-I secretion in ER-retention conditions when ascorbate is deficient (Miller and Grosel, 2020). Collagen-I and ERp29 may interact at one of the several –(F,Y)-(F,Y)- or –(F,Y)-X-(F,Y)- motifs that are present in collagen-I’s primary structure (Barak et al., 2009). Interestingly, no naturally occurring mutations are documented in any of these potential ERp29 recognition sites. Instead, many of the documented disease-causing collagen-I mutations occur in glycine residues that appear at roughly every third residue in the primary structure and likely interfere with proper formation of mature collagen’s fibrillar structure. Future study is not only required to pinpoint the precise binding site of ERp29 to collagen-I, but more importantly to expand on ERp29’s stabilizing function in regard to collagen-I formation.

### ERp29 as a Regulator of Protein Assembly

The discussion thus far has focused on ERp29’s role in promoting the biogenesis of clients, including trafficking of channels or transporters to the plasma membrane and secretion of proteins with primarily extracellular functions. Some of these considerations suggest a role for ERp29 in regulating assembly of clients into higher order oligomers, which is discussed in more detail here.

### Connexin 43 (Cx43)

Connexins are integral membrane proteins that play a critical role in intercellular communication by forming gap junctions that allow ion transfer between adjacent cells, importantly Ca^2+^ (Goodenough and Paul, 2003), and have therefore been implicated in many human diseases (Table 1; Pfenniger et al., 2011). One of the most ubiquitously expressed Connexin isoforms is Connexin43 (Cx43), which is highly expressed in the heart and may play an important role in diseases of myocardial conduction (Laird, 2008; Das et al., 2009). When forming a Cx43 channel at a tight junction, each neighboring cell contributes a Cx43 hemichannel that is composed of 6 Cx43 subunits. In fact, it was initially thought that mutations of the Cx43 gene, *GJA1*, would be lethal, given its importance for cardiac function; however autosomal dominant mutations in *GJA1* were recently linked to oculodentodigital dysplasia (ODDD), a developmental disorder with some myocardial implications (Paznekas et al., 2003; Laird, 2008). In addition to forming gap junctions by the interaction of hemichannels from adjoining cells, Cx43 plays a critical role in resisting oxidative stress (Kar et al., 2013; Zhao et al., 2017; Chen et al., 2018) and has been shown to directly interact with beta-catenin (Spagnol et al., 2018). Therefore, understanding the critical factors that underlie Cx43 biogenesis could provide key insights into the underlying pathology of ODDD and related diseases.

ERp29 promotes the assembly of functional hexameric Cx43 hemichannels in the Golgi of HeLa cells via stabilization of Cx43 monomers in the ER and through their transport to the Golgi (Das et al., 2009). Of relevance to this discussion, treatment with 4PBA improved the ability of HeLa cells to process overexpressed Cx43 into hemichannels; such overexpression of Cx43 would otherwise saturate the processing system and result in premature oligomerization of the monomers in the ER (Asklund et al., 2004; Das Sarma et al., 2005, 2008). As 4PBA increases ERp29 protein expression (Suaud et al., 2011a), further experiments demonstrated that increased ERp29 expression inhibited the premature oligomerization of Cx43 monomers in the ER (Das et al., 2009). Furthermore, interfering with ERp29 function led to accumulation of Cx43 in the Golgi, decreased Cx43 abundance at the plasma membrane, and impaired gap junction communication as measured by intercellular transport of fluorescent dye (Das et al., 2009). Taken together, these data suggest that ERp29 stabilizes monomeric Cx43 in the ER and prevents premature oligomerization, and thus plays a critical role for the proper biogenesis and assembly of functional Cx43 hemichannels. Interestingly, the assembly of Cx43 hemichannels in the Golgi requires formation of inter-subunit disulfide bonds, a process that ERp29 cannot itself catalyze since it lacks the thioredoxin domain that is typical of PDI-like proteins (Bao et al., 2004; Mkrtchian et al., 2008). This characteristic of ERp29 may suggest that it has evolved a specific role in stabilizing proteins until later processing and/or disulfide bond formation.

### Proinsulin

ERp29’s stabilization of monomeric Cx43 could indicate a broader role for ERp29 in stabilization of immature proteins during trafficking. Of particular interest, ERp29 overexpression appears to stabilize and increase the abundance of intracellular Proinsulin in models of pancreatic islet β-cells (Viviano et al., 2020). Like Cx43, Proinsulin is suggested to form hexamers in the Golgi (Musil and Goodenough, 1993; Kiselar et al., 2011; Haataja et al., 2013), which are subsequently cleaved into insulin and C-peptide by furin-like convertases in the Golgi or later compartments (Smeekens et al., 1992; Nishigori et al., 1996). Our group has recently demonstrated, using both MIN6 and INS1 pancreatic beta cells, that ERp29 associates with Proinsulin, that overexpression of WT ERp29 increases the intracellular abundance of both Proinsulin and mature Insulin, and that depletion of ERp29 decreases the intracellular abundance of Proinsulin (Viviano et al., 2020). On the other hand, overexpression of the C157S ERp29 mutant increases the abundance of intracellular Proinsulin but does not result in a corresponding increase in mature Insulin (Viviano et al., 2020). These data are consistent with the hypothesis that ERp29 stabilizes intracellular Proinsulin, and that ERp29 promotes Proinsulin cleavage to Insulin and C-peptide in a manner that is dependent on its C157 residue. Furthermore, ERp29 appears to associate with both Proinsulin and the COP II vesicle precomplex component Sec24D (Viviano et al., 2020), likely as part of larger complex as Sec24D is cytoplasmic and ERp29 and Proinsulin are within the ER lumen. These data may suggest a role for ERp29 in promoting Proinsulin’s inclusion in COP II vesicles, possibly via KDEL-R (Grumbach et al., 2014; Bikard et al., 2019; Viviano et al., 2020). It is thus possible that ERp29 facilitates Proinsulin processing in a manner that parallels that of Cx43. Specifically, Ep29 may prevent premature oligomerization of Proinsulin in the ER and delay the formation of Proinsulin hexamers until Proinsulin reaches the Golgi.

ERp29’s potential role in the resolution of ERS and UPR is also of particular interest when considering the pancreatic β-cell. A healthy ER environment is critical for the maintenance of functional Islets of Langerhans, and ERS appears to contribute to the development of Type II Diabetes (T2D) (Sundar Rajan et al., 2007). Additionally, mutations in the stress responsive ER resident protein, PERK, previously mentioned as working in concert with ERp29 to regulate the UPR, cause Wolcott-Rallison Syndrome, which among other things, results in permanent neonatal diabetes characterized by low β-cell mass (Senée et al., 2004; Gupta et al., 2010). It was previously hypothesized that this phenotype resulted from an impaired ER stress response in the β-cell, as PERK has been predominantly implicated in UPR and ERS (Harding et al., 2001). However, recent evidence suggests that low β-cell mass in the absence of functional PERK is a result of impaired ER→Golgi transport, resulting in the accumulation of Proinsulin in the ER and suggesting a role for PERK in Proinsulin ER exit (Gupta et al., 2010). Additionally, PERK was demonstrated to co-precipitate with Proinsulin (Gupta et al., 2010), and, as previously mentioned, co-precipitates with ERp29 as well (Farmaki et al., 2011). As such, PERK’s role in maintaining β-cell function appears to also implicate it playing a role in trafficking, perhaps in addition to its more studied role in ERS and UPR ER. Thus, it is possible that ERp29 and PERK act in concert to facilitate Insulin biosynthesis in pancreatic β-cells.

## Discussion

ERp29 is hypothesized to facilitate the functional processing and maturation of a diverse range of client proteins implicated in diseases of protein misfolding or trafficking. However, there are still critical gaps in our understanding of ERp29’s many and diverse functions, especially as it relates to these diseases of protein misfolding or mistrafficking. Because ERp29 is a member of the PDI/thioredoxin family, but has no thioredoxin activity, it is hypothesized to function as an escort that facilitates client export from the ER. While there is still much to learn regarding ERp29’s mechanisms of action, recent data suggest that ERp29 also promote the inclusion of client proteins in COP II vesicles for ER→Golgi transport. As ERp29 is an ER luminal protein and COP II machinery is cytosolic, it is logical to hypothesize that an integral membrane intermediary must facilitate ERp29’s role to recruit cargo to COP II vesicles. In fact, our recent data suggest that the KDEL-R is one such potential intermediate, as it works in concert with ERp29 to promote the Golgi processing of ENaC. As the KDEL-R continuously cycles between the ER and Golgi via COP II and COP I vesicles, respectively, KDEL-R is an attractive candidate to mediate ERp29’s association with COP II. However, the ubiquity of the role of KDEL-R in promoting ERp29-facilitated cargo recruitment for COP II vesicles requires further investigation, and there may be other proteins involved in the proposed ERp29/KDEL-R/COP II complex yet to be identified. Furthermore, ERp29 appears to also stabilize immature proteins, preventing premature oligomerization of Connexin43 (Cx43) and increasing the intracellular abundance of Proinsulin. ERp29’s role in protein stabilization prior to full maturation also requires further interrogation.

ERp29 likely forms large complexes in the ER lumen, interacting with client proteins, other chaperones, vesicle adaptors, and receptors such as KDEL-R. The domain structure of ERp29 is well-delineated, however the contribution of individual, evolutionarily conserved amino acid motifs to specific protein–protein interactions has not been fully investigated. ERp29’s C-terminal KEEL ER retention sequence is one such motif. Importantly, this ER retention motif is thought to be “leaky” with regards to ER retention, in that proteins with KEEL motif are more likely to transverse the Golgi and be secreted compared to those with the classical KDEL. These data suggest that ERp29 may have additional function distal to COP II vesicle fusion with the Golgi membrane; in other words, ERp29 may also function as a chaperone in the Golgi or later compartments. However, potential function(s) for ERp29 in Golgi have yet to be thoroughly interrogated, as well as similar Golgi roles of other ER-resident chaperones with “leaky” KEEL motifs.

Additionally, ERp29 lacks the classical thioredoxin domain of most PDI-like ER chaperones; in its place is a single, evolutionarily conserved cysteine, Cysteine157, which is the only cysteine in the protein structure. While C157 does not have a predicted function in client interaction, introducing a C157S mutation into human ERp29 leads to the loss of function. Additionally, this Cysteine is located at the base of ERp29’s D-domain, which is essential for client binding and processing, and mutation of this Cysteine is predicted to change the hydrophobicity characteristics of the linker between b-domain and D-domain, which triggers structural instability of the D-domain (Hermann et al., 2005). Interestingly, ERp29 in rats and humans has a longer linker between its two structural domains as compared to mouse ERp29 and *Drosophila* Wind, which could result in higher structural mobility of two domains in respect to each other in the rat and human proteins. Future studies should aim to interrogate the mechanistic importance of both these conserved motifs, as well as effect variations between species, including the variations in linker length, may have on ERp29 function.

ERp29 may also play a critical role in ER stress and the unfolded protein response (UPR), however, its mechanism of action in this setting is similarly not yet fully elucidated. Like many ER chaperones, ERp29 expression is upregulated during ERS and UPR. Although increased ERp29 expression is not sufficient to induce ERS, its relationship with key ERS sensor proteins, PERK and BiP, suggest that it may enhance or otherwise regulate UPR and ERS. However, ERp29’s relationships with other regulators of ERS has not yet been investigated in depth, nor has the downstream effect of its relationships with PERK and BiP. It is therefore important to further interrogate the mechanistic role of ERp29 in ERS. It is important to note that ERS, its resolution, and its potential to lead to apoptosis are relevant to a broad spectrum of diseases ranging from CF and T2D to cancer and neurodegeneration. As 4PBA upregulates expression of ERp29, apparently indiscriminate of tissue type, the improved processing and maturation of a number of proteins with 4PBA treatment implicate ERp29 and its increased expression levels as a potentially common mechanism of action. Therefore, it is logical to investigate ERp29’s relationship with other proteins whose processing is improved by 4PBA treatment, including A1AT; ATP-binding cassette proteins ABCA1, ABCA3 and CFTR (ABCC7); bile salt export pump, BSEP; creatine transporter, CTR-1; and monocarboxylate transporter, Met8, among others.

In summary, ERp29 is a non-classical molecular chaperone of the ER that appears to have diverse roles in maintaining functional and efficient protein processing. Although ERp29 is a PDI-like protein, it lacks a thioredoxin domain and is therefore likely redox inactive. In this way, ERp29 is unique, and functions as a chaperone or escort of immature client protein, stabilizing clients and facilitating their transport from the ER to the Golgi for maturation. ERp29 is most highly expressed in secretory cells, and likely facilitates efficient processing of precursors to secreted proteins, including Proinsulin, Thyroglobulin (Tg), and perhaps immature α1-Anti-trypsin (A1AT). However, ERp29 is also ubiquitously expressed in non-secretory tissues and across organ systems, aiding the maturation of non-secretory membrane proteins as well, including Cx43 hemichannels or CFTR and ENaC in epithelial tissue. ERp29 also appears to play a role in cellular stress processes, including ERS and UPR; and even has a demonstrated neuroprotective effect. Thus, alterations in ERp29 function may have implications for cancer cell survival, β-cell demise in T2D, and neuroprotection in Alzheimer’s and Parkinson’s diseases. Its significant role in secretory tissue makes ERp29 a potential target for treatment of A1AT deficiency, diseases of thyroglobulin processing, and Type 2 Diabetes. ERp29 also rescues the trafficking of the most common CF-causing mutant, F508del, resulting in functional CFTR protein at the membrane, and promotes the maturation of functional ENaC. To summarize, ERp29’s many and diverse roles, as well as its regulation by 4PBA, make it an attractive clinical target for a wide range of diseases of protein folding.

## Data Availability Statement

The original contributions presented in the study are included in the article/supplementary material, further inquiries can be directed to the corresponding author.

## Author Contributions

All authors contributed equally to this manuscript. Figures 2, 3 were created by MB using BioRender.com.

## Conflict of Interest

The authors declare that the research was conducted in the absence of any commercial or financial relationships that could be construed as a potential conflict of interest.
